# How Pediatricians Can Deal with Children Who Have Been Sexually Abused by Family Members

**DOI:** 10.5402/2011/258640

**Published:** 2011-12-08

**Authors:** Ruth Wolf

**Affiliations:** Interdisciplinary Department of Social Sciences, Bar-Ilan University, Ramat Gan 52900, Israel

## Abstract

The present paper discusses children who have been the victims of sexual abuse in their own family. It focuses on the special role of pediatricians and medical staff in identifying such children and providing them with initial assistance by reporting the situation to the authorities. The first part of the paper surveys the short- and long-term effects of childhood sexual abuse, including the physical and emotional impact of sexual exploitation and severe neglect. This section discusses the symptoms shown by abused children, and how they express and deal with their trauma. It is extremely important for pediatricians to be sensitive to the possibility of patients being abused at home, as this is an area still largely regarded as a societal taboo. Also included in this section a discussion of the effects that are manifested when the victim has grown to adulthood, such as personality disorders. The second part of the paper deals with how pediatricians must act when they encounter such a situation in which they suspect sexual abuse in the family. They should strive to identify the problem and bring it to the attention of the authorities. Discovery of the problem is the most vital part of the path to the victim's recovery. The paper also discusses the aspect of treatment, advising doctors who encounter this problem on ways of dealing with it.

## 1. Introduction

The present paper discusses the important role that pediatricians play in locating and diagnosing children at risk. Children are the most common prey for sexual exploitation. Much abuse of this sort takes place within the child's own family. It is precisely those people that the child relies on, and from whom he expects to receive understanding, support and love, that turn their back on one of the most important values in any society—protecting the young. 

According to American law, the definition of sexual abuse “…includes sexual assault and sexual exploitation. Sexual assault includes sex acts with children, intentional masturbation in the presence of children and child molestation. Sexual exploitation includes preparing, selling or distributing pornographic materials involving children, performances involving obscene sexual conduct and child prostitution” (P.C. 11165.1). 

Since the person who abuses the child is so often a member of his own family, the children who undergo these painful experiences can generally only be identified by people outside the family. Often the best place for finding such children is the doctor's office or medical center they are attending. Thus, it is the pediatricians who are most likely to be able to rescue these young children from the continuing abuse they are being subjected to, and provide them with support and assistance.

## 2. The Extent of the Phenomenon

According to Somer [1], for example, one in seven children in Israel is sexually molested by a family member. Most of these, about 80%, are girls, while the minority who are male are generally abused by members of their own sex. A broad study was conducted by Pimlott-Kubiak and Cortina [2] in the United States, in which 16,000 subjects, both men and woman, were asked about different types of abuse, 18% of the women and 3% of the men described an experience of sexual abuse, while 54% of the men and 40% of the women had undergone physical abuse at least once during their childhood. Finkelhor [3] also found a preponderance of women who have experienced sexual abuse, with one and a half to three reports about girls for every report about a boy.

It is not clear how many girls compared to boys are abused (American Humane Association, 1981) but research highlights that these cases of abuse include not only the abuse of young girls by older men. It is important to note that this phenomenon is apparent in both genders: men as well as women. Abusers of boys are usually men. Statistics note that approximately 14-15% of girls and approximately 14–24% of boys are abused by women. 

A study in Israel notes that pediatricians classified the abusive events that they encountered according to categories and noted their severity. It was found that they considered sexual abuse as presenting the greatest risk to a child after educational abuse, learned delinquency, and physical abuse, followed by physical neglect, psychological neglect, and educational neglect [4, page 149]. It was also found that the doctors' reasons for not reporting cases to the police have to do with their perception that police officers do not have the tools or skills necessary to handle the issue of child abuse. The main reason for not reporting cases of known child abuse to the authorities is the doctors' desire to ensure the welfare of the child [5].

The purpose of this paper is, therefore, to draw the attention of pediatricians to the importance of identifying these abused children so as to rehabilitate them and allow them to develop properly, as the sexual abuse of children damages not only their present but their future development as well [6–8].

Studies [9] have shown that the younger the abused child, the greater the likelihood that the abuser is known to the child or even a member of his family. An analysis of the data by Bentovim et al. [10] revealed that in 23% of the cases where a child was sexually abused by a family member another child in the same family was also abused and in 12% of the cases there were three or more abused children in the same family. Thus, when pediatricians or medical staff encounter an abused child, they need to alert the authorities to check the other children in the family for possible abuse as well.

According to the testimonies of pediatricians that met with children who were sick or had come in for check-ups, they sometimes encountered children who appeared dejected, who lacked joy, who seemed to be in a permanent state of sadness, and some of them told them very painful stories. There is no evidence in the literature of any identifying characteristics of the families in which children are exposed to sexual abuse and exploitation. Some come from the richest homes and some from the poorest, some religious and others secular, and some from a high social class and other from a low social class. No common denominator has been found among the homes in which these dark crimes take place against those who are meant to be according the appropriate conditions for growth and development [1, 8, 11].

There has recently been much greater media exposure of incidents of sexual violence against children. Most probably this is not due to any increase in acts of incest and sexual exploitation of children but rather to a greater involvement of the media in bringing such cases to our attention. We hear about children who are used as sexual slaves by their parents, mothers who use their offspring to satisfy their own needs, and other family members who rob children of their innocence. Such violence, which is both physical and emotional, is incomparable in its profound effects, which continue from one generation to the next.

Most countries have laws requiring educators to report abuse of children, and this sometimes leads to a court order removing the children from the family. But even in these cases the damage has already been done and the child has developed a way of life in which he approaches the world with hostility, disappointment, and a total lack of trust. A family in which sexual abuse of children occurs cannot serve as a means of support for the children, and a child in such a situation generally cannot tell anyone in the family, even those who are not the abuser, about what has occurred. In many cases the abuser threatens the child with further harm if he dares to tell anyone about the abuse, causing his to keep away from other people in order to conceal the terrible secret.

## 3. Difficulties Involved in Studying the Topic

It is difficult to identify young children who are being sexually abused. Children under the age of 6 have difficulty communicating what has been happening to them. Occasionally the media mentions that, after the offender has been caught, the victim's parents or relatives reported that the child had protested against being left with the babysitter or relative who molested them. Nevertheless, it is possible to discover from the child's actions that he is being abused, due to the phenomenon of reenacting the trauma. When children play, they tend to reenact experiences of their everyday life, and so the evidence for traumatic experiences at this age can be found by watching them at play and noticing their reenactment of behavioral patterns characteristic of aggressors and victims. 

Sometimes a child will also speak in a way that betrays the experience he has undergone. If, for example, a child demonstrates knowledge of sexual matters that is inappropriate for his age, this should immediately arouse suspicion that he has been exposed to sexual abuse of this sort. If a child tells his doctor about events involving sexual seduction, it is unlikely that he is making it up. Young children rarely say that their playmates have been seducing them sexually, except possibly in play. 

Terr [12] noted that inappropriate sexual behavior in young children is a clear sign that this sexual knowledge was thrust upon the child by some external source. Examples of such behavior are playing sexual games with dolls, repeated masturbation in ways that are very unusual in young children, such as introducing objects into their vagina or anus, or the use of seductive expressions during doll play with other children. 

One of the important difficulties involved in studying the sexual abuse of children within the family is that the society as a whole follows the victims in suppressing any thought of it. The victims' inability to bear the trauma often leads them to suppress the whole issue, and many of them find it so hard to deal with that they construct defenses to cope, such as forgetting, repression, and denial. The victims' use of these defense mechanisms makes it very difficult for others to discover the truth of what happened. Some of the victims “remember” the trauma at a much later date, bringing about a situation in which both clinical and research psychologists must confront the difficult question of whether patients' “recovered memories” of childhood sexual abuse are true memories of traumatic past events or false memories based on oedipal fantasies. Among the many researchers discussing this question are Somer [1] and Hepburn [13]. 

Even mature children who manage to deal with their traumatic memories of sexual abuse (often by pretending they have an ideal family life) try to prevent others from finding out about it. Feelings of guilt, horror, rage, and helplessness lead the victims to the use of defense mechanisms and other coping methods to avoid facing their own questions about what has been happening to them. Childhood sexual abuse within the family is thus seriously underreported, making it very difficult to find out its exact extent.

## 4. The Medicine Center's Role

Often the first place that children who are abused by a family member can be helped is within the medical center they are attending. When a physician notices unusual signs in a child—sadness, separation from the group, a sharp decline in cognitive achievement, a preference for being alone, attention problems, depression, lack of energy, social problems, secretiveness—this is a clear indication of some serious disturbance in the child's life. Although these signs show that the child is experiencing a severe problem, it is possible that the cause has nothing to do with sexual abuse. Nevertheless, since the statistics show that sexual abuse is so widespread and since these symptoms do occur in most children who have been sexually abused, each case should be carefully considered to see what lies behind the symptoms.

There are instances in which a pediatrician examines a young victim whose parents present her as incoherent, mentally ill, or a dreamer in order to prevent her from exposing her assailants. The problem is reinforced when there is a legal guardian for the child, a parent or other individual whose protection he is living under. In this case, the victim is also dependent on him and this is liable to present a false picture of the physical and emotional situation in order to hide the abuse. The doctor is the one who generally comes into contact with the victims and their legal guardians. He is the one who physically examines the child and can also refer to his mental state within the family system, usually for a short amount of time or at a critical juncture.

According to the Penal Code in California Law, noted by the California Department of Social Services in Child Abuse Prevention, physical indicators of sexual abuse in the child include “bruising around genital area; swelling or discharge from vagina/penis; tearing around genital area, including rectum; visible lesions around mouth or genitals; complaint of lower abdominal pain; painful urination, defecation” (page 8). 

Research shows that people who have experienced sexual abuse complain more than other patients about gastronomical issues [14, 15]. These researchers also found that women who experienced sexual abuse in their childhood visited doctors more than other women. They also displayed more depressive symptoms. 

Children's unusual behavior sometimes testifies to problems and distress, which may be connected to sexual exploitation beyond the child's physical signs and symptoms.

This has often helped uncover a terrible, threatening secret that the child is afraid to tell anyone about and thus can only hint at. In older children, who are more able to write, their writings can open up a window on their psyche for the sensitive pediatrician. In younger children, even the task of painting can enable the child to develop appropriately in the cognitive, emotional, and social spheres. They must therefore be alert to the signs hinted at by children's behavior, even if it is caused by events that are unconnected to the medical realm.

## 5. The Traumatic Effects of the Abuse

This paper attempts to explain the harm done to the child in the present and in the future as a result of being the victim of sexual abuse within the family. Most such incidents are not revealed even at the present time. There is a good deal of discussion in the literature [3, 7, 11, 13, 16–27] about the aftereffects of trauma due to rape, sexual abuse, and family violence. It has been found that victims tend to develop posttraumatic stress syndrome. Livingston [21] also found that some victims developed psychotic symptoms, depression, somatic complaints, and adjustment problems.

Neumann et al. [28] conducted a meta-analysis of 38 studies of the relationship between a history of childhood sexual abuse and psychological problems in adult women. The symptoms found among these women included “anxiety, anger, depression, revictimization, self-mutilation, sexual problems, substance abuse, suicidality, impairment of self-concept, interpersonal problems, obsessions and compulsions, dissociation, post-traumatic stress responses, and somatization” (ibid., page 6), all of which were significantly associated with sexual abuse. 

All violence is damaging, but when children are involved it is considerably worse. All expect adults to support children and to help them grow and develop. Sexual violence against children causes the greatest damage. It tears apart the individual's sense of intimacy and subverts one's sense of oneself. It creates feelings of shame, guilt, and precocious maturity. It is not merely a physical injury. It also damages all of the individual's psychological and physical functions, both as they exist in the present and as they are meant to serve the individual in the future [3, 16, 23, 24, 26].

A major problem stemming from incest and sexual exploitation involves the abuser. As mentioned, many instances of sexual violence occur within the immediate family. Although any form of sexual abuse is damaging to a child, it is severely destructive when it is perpetrated by a member of the child's own family. Victims of sexual abuse on the part of a parent or other close relative perceive the damage done to them as being greater and complain of more severe symptoms, than victims of sexual abuse on the part of strangers [24, 29, 30]. It is expected that a parent, grandparent, or uncle, for example, will demonstrate affection and support the child. When such a person abuses the child instead, it creates feelings of insecurity and pain and a sense of disintegration.

As Erikson [31, 32] pointed out, the early childhood years are the ones in which the child constructs his way of life. If he is supported, nurtured, and loved, he will develop a positive outlook of the world. A child who is abused, in contrast, generally adopts a way of life without trust in the adult world. According to Erikson, it is the child's daily interaction with his family that affects his view of life. The mutual relationships within the family and the child's place in it have a major effect on the way of life the individual will adopt. Children who have to deal with painful experiences in which family members use them—their bodies and their psyches—as a way of satisfying their own drives, without consideration for the children's autonomy, lose the sense of self-esteem that is essential for proper development.

The idea that children who have undergone sexual trauma lose their sense of self-esteem can also be found in research done by Kinston and Cohen [33]. Moreover, Guillon et al. [34] found that victims of sexual abuse had a lower degree of self-esteem than those who had not undergone such abuse, with female victims having lower self-esteem than male victims. 

In addition, as noted by Gunderson and Sabo [20], later disorders linked with the trauma of sexual abuse resemble PTSD (posttraumatic stress syndrome) and include substance dependency, psychosomatic complaints, and general psychological distress. Such disorders can arise from the outbreak of unbearably painful memories. It is possible that the victims develop defense mechanisms in an attempt to erase these memories and prevent the trauma from affecting their lives. Sometimes a victim may project his anger and stressful feelings onto others. These painful feelings may emerge from their repressive state at any stage, and they can have extremely painful manifestations.

People often return to what is familiar, no matter how painful, out of the sense that it is easier to deal with what is known. Aggressor-victim relationships are thus liable to be repeated in the future, with the victim becoming the aggressor. This damages the children of the former victims by making them victims in turn. Livingston [21] explains this phenomenon with the notion of “trauma addiction.” They note that victims of childhood sexual abuse often become prostitutes, while victims of physical abuse may become self-destructive. A study of boys who had been sexually abused and later became abusers themselves was conducted by Bentovim [35]. The study found that sexually abused boys who lived in a “climate of violence” and had poor care were liable to abuse other children in turn. 

Brown and Anderson [36] found a link between childhood sexual abuse and personality disorders severe enough to require psychiatric hospitalization. Links have also been found between childhood abuse and later criminal behavior [37–40].

## 6. Body Memory

Hepburn [13] uses the phrase “body memories” to describe her theory that it is the body that remembers physical traumas, even those that the individual is unable to recall consciously. In this view, such memories are imprinted on the body and manifested in psychosomatic symptoms like convulsions, pelvic contractions, and paralysis of various parts of the body [41]. This shows how the physical and psychological trauma of childhood sexual abuse can affect the individual over the long term. According to these theorists, the victims of sexual abuse have a tendency to suffer from depression, anxiety, impulsive behavior, and suicide attempts.

As Hepburn [13] points out, the shame, confusion, and feeling of unsuitability remain with the child even after he has grown up. And when the time comes at which it is expected that the adult will be able to make use of his sexuality for his psychological and physical well-being and for contributing to the next generation, these emotional memories will come flooding back, causing him to reexperience those early feelings and thus damaging his ability to function sexually as an adult [23, 42].

Putnam [43, 44] claims that there is yet another phenomenon that is often found among victims of sexual abuse, which is linked to the repression of early memories, namely, dissociative identity disorder, which involves memory distortion and personality splitting. This phenomenon, which is a result of massive denial mechanisms, is said to occur in trauma victims who are unable to bear their memories.

## 7. How Pediatricians Can Support Victims of Sexual Abuse

The main purpose of this paper is to present a harsh phenomenon, which is usually kept under wraps as a “terrible dark secret.” This situation could be ameliorated if the people who enter into interactions with children and youth, and who have the skills and the tools to diagnose them, would pay more attention to the symptoms and behavior described above. It is important to help pediatricians and other members of the medical staff to look out for behavior that could be indicative of a child who is being sexually abused. The task of the pediatrician in such cases is mainly to call in the proper authorities to deal with the matter, while offering the child initial counseling and immediate help. 

This assistance is very important because the victim is powerless and has no one else to depend on. The pediatrician's responsibility in cases like this is very great. They are the ones who can bring about change and break up the terrible situation that the victim is caught in. Pediatricians are expected to turn to the authorities if they suspect a child is being sexually abused. It should be emphasized that this applies even when there is only a suspicion of abuse. They are not meant to occupy themselves with real detective work but rather to try to examine the issue and report it to the authorities. It is reasonable to assume that the authorities will direct these children to supportive frameworks and appropriate psychological counseling.

The American law notes that Child abuse must be reported when one who is a legally mandated reporter “…has knowledge of or observes a child in his or her professional capacity, or within the scope of his or her employment whom he or she knows or reasonably suspects has been the victim of child abuse or neglect…” (P.C. 11166(a)). “Reasonable suspicion” occurs when “it is objectively reasonable for a person to entertain such a suspicion, based upon facts that could cause a reasonable person in a like position, drawing when appropriate on his or her training and experience, to suspect child abuse.” (P.C. 11166(a)(1)). Although wordy, the intent of this definition is clear: if you suspect, report (page 4-5) [45].

## 8. Conclusion

The necessary conclusion to be drawn from the analysis of this case is that society must adopt a strong and clear policy condemning this sort of abuse. Those of us who work as psychologists have the task of drawing society's attention to this phenomenon, and particularly of alerting people who deal with children to keep their eyes open constantly for any signs that these children might be suffering from abuse. Great sensitivity is needed to locate and diagnose these deviant phenomena, and when such cases are discovered immediate assistance must be provided for the victims so as to ensure that they will be able to develop as well as possible in the future. 

This change can be implemented if doctors in formal institutions, especially pediatricians, family doctors, emergency room doctors, and those who interact with children, pay attention to these signs and the symptoms that we mentioned above. It is important to mention that the role of the doctors is mainly to discover this phenomenon and to administer “first aid”—psychologists, psychiatrists, and social welfare workers are meant to continue the treatment for those harmed children. 

This is crucial both to prevent the abuse from continuing and to enable the victims to obtain proper treatment. The damage suffered by victims of childhood sexual abuse is immense. It is precisely the conspiracy of silence that generally surrounds this painful issue that prevents pediatricians from locating these cases at an early stage. Since the victims use various defense mechanisms, including repression, to hide the intensity of the pain and damage, our goal as human beings and as pediatricians must be to help them heal their psyches and allow them to begin a new journey to a better life.
